# Are you tired of “us?” Accuracy and bias in couples’ perceptions of relational boredom

**DOI:** 10.1177/02654075231168141

**Published:** 2023-04-03

**Authors:** Kiersten Dobson, Sarah C. E. Stanton, Rhonda N. Balzarini, Lorne Campbell

**Affiliations:** 1 Department of Psychology, 6221 University of Toronto at Mississauga,Mississauga, ON, CA; 2 Department of Psychology, University of Edinburgh, Edinburgh, UK; 3 Department of Psychology, 7174 Texas State University, San Marcos, TX, USA; 4 Department of Psychology, University of Western Ontario, London, ON, CA

**Keywords:** relational boredom, tracking accuracy, directional bias, relationship quality, response surface analysis

## Abstract

Relational boredom is an important cognitive-emotional experience that is understudied in the relationship maintenance literature. In three dyadic studies, we investigated accuracy and bias in partners’ perceptions of each other’s relational boredom, and how accurate and biased boredom perceptions were associated with relationship quality. Results revealed that, overall, partners tended to overestimate—but accurately track—each other’s relational boredom across the features that comprise relational boredom and across time. Additionally, when people accurately perceived their partner experiencing high levels of boredom, they reported lower relationship quality; in all other cases, one’s own relationship quality was preserved. Furthermore, when people accurately perceived their partner experiencing high levels of boredom, their *partner* also reported lower relationship quality, while the partner’s relationship quality was consistently preserved when the perceiver was accurate at low levels of boredom or overestimated. These findings have important implications for how couples navigate boredom and maintain long-term relationships.

## Introduction

Romantic couples face several challenges when attempting to maintain satisfying, long-term relationships. One subtle but pervasive challenge couples are likely to encounter is *relational boredom*, the tendency for partners to feel “tired of” and unstimulated in their relationship (Harasymchuk & Fehr, 2010; 2013). Relational boredom is characterized by a variety of negative emotions (e.g., lack of excitement, frustration) which can lead to declines in relationship quality (e.g., Reissman et al., 1993). In fact, one clinician concluded that “boredom is perhaps the most common denominator of all marital problems” (Venditti, 1980, p. 65). Thus, experiencing boredom is clearly problematic for relationship success; however, how partners *perceive* one another’s boredom remains an open question. Are perceptions of a partner’s relational boredom rooted in reality? What happens to the relationship if those perceptions are inaccurate? Or, to the perceiver’s credit (but likely also to their chagrin), what happens if those perceptions are correct?

Guided by theoretical models of boredom, interdependence, and accuracy and bias in interpersonal perception, we investigated whether romantic partners accurately track the pattern of each other’s relational boredom and whether they over- or underestimate each other’s boredom (Research Question 1; RQ1). We also examined whether partners’ accurate and biased perceptions of relational boredom were associated with relationship satisfaction, commitment, and trust (RQ2). We tested these ideas in two dyadic cross-sectional studies (Studies 1 and 2), and a 21-day dyadic daily experience study (Study 3).

### Relational Boredom

Boredom is a common experience and represents both a low- and high-arousal cognitive-emotional state wherein people can feel simultaneously understimulated and restless (Westgate, 2020). In their prototype analysis of relational boredom, Harasymchuk and Fehr (2010, 2013) found that when romantic partners describe what relational boredom feels like, they report low-arousal, low-pleasure emotions (e.g., feeling unexcited or tired) as well as high-arousal, low-pleasure emotions (e.g., feeling frustrated or anxious). These negative emotions stem primarily from a lack of novelty or stimulation within the relationship (e.g., unmet expectations; Harasymchuk et al., 2021), or from external causes (e.g., attractive alternatives; Harasymchuk & Fehr, 2010, 2013). Unsurprisingly, then, individuals experiencing relational boredom also tend to report lower investment and satisfaction in the relationship and a greater willingness to engage in infidelity (e.g., Gillen et al., 2012).

Currently, researchers know much about relational boredom experiences at the individual level. Intimate relationships, however, are inherently interdependent, meaning that the thoughts, feelings, and behaviours of one partner are informed by and influence the thoughts, feelings, and behaviours of the other partner (Kelley et al., 2003). As an example, imagine that Meredith and Derek are a romantic couple. Interdependence theory posits that we must consider how Meredith’s relational boredom is related not only to her *own* satisfaction and commitment, but also to *Derek’s* satisfaction and commitment, and vice versa. In the present research, we test this mutual influence, especially in terms of how Meredith’s *perceptions* of Derek’s relational boredom—contrasted with Derek’s reported experiences of boredom—are related to her own and Derek’s relationship outcomes.

Boredom conveys important information for partners, primarily that continuing the current course of action “as is” is neither fulfilling nor worthwhile (see Westgate, 2020). How romantic partners obtain and deal with this information, then, plays a key role in the future success of the relationship. However, partners experience opposing needs in relationships that may drive them to be accurate or inaccurate in their perceptions of each other’s boredom. According to risk regulation theory (Murray et al., 2006; 2008), partners must balance the need for closeness, dependence, and intimacy with the need to maintain distance to protect the self from hurt and rejection. When regulating risk, individuals appraise their partner’s feelings about the relationship and their likelihood of being rejected, actively gathering the information needed to respond in the future. Without making judgments of their partner’s thoughts and feelings, individuals may not know whether to enact closeness-promoting behaviours that are “worth the risk,” or to distance themselves from their partner to avoid being hurt. We argue that appraising a partner’s relational boredom should be a regular part of navigating intimate relationships over time.

### Accuracy and Bias in Relationship Perceptions

Partners’ perceptions of each other tend to be accurate and rooted in reality; however, these perceptions often also contain some degree of bias (Fletcher & Kerr, 2010; West & Kenny, 2011). Collectively, extant literature points to three main forms of perceptual accuracy and bias. *Tracking accuracy* represents how much perceivers can correctly detect the pattern of a partner’s responses (e.g., across items of a scale, across time, etc.). *Directional bias* (also called *mean-level bias*) reflects how much perceivers generally over- or underestimate their partner’s responses. Lastly, *assumed similarity* (also called *projection*) represents how much perceivers’ judgments of their partner are predicted by their own beliefs (i.e., the extent to which they project their own beliefs onto their perceptions of their partner). In the current research our primary interest involved examining tracking accuracy and directional bias. Nonetheless, we account for the effects of assumed similarity in our models as it is a key component of accuracy and bias models and because in doing so tracking accuracy reflects direct accuracy (West & Kenny, 2011), or accuracy when projection is taken into account. These forms of accuracy and bias emerge in perceptions of partners’ ideal standards (Campbell et al., 2013), commitment (Overall & Hammond, 2013), emotions (Clark et al., 2017), regard during conflict discussions (Overall et al., 2012), approach and avoidance motives (LaBuda et al., 2019), sexual desire (Muise et al., 2016), and sexual advance and rejection behaviours (Dobson et al., 2018, 2022). This literature points to the important interplay of accuracy and bias within intimate relationships and the relationship consequences of accuracy and bias.

Partners are likely to accurately track the pattern of each other’s relational boredom. Accurate appraisals of one’s partner and relationship are an important component of effective risk regulation (Murray et al., 2006), as they balance the need to protect the self with the need to enhance and develop the relationship. Therefore, partners should be attuned to relationship-threatening feelings, including boredom. Returning to our hypothetical couple, Derek should be motivated to correctly detect Meredith’s relational boredom because doing so will not only help him understand when Meredith’s needs are not being met but may also protect him from continuing to invest in a relationship that Meredith now believes is unfulfilling. Additionally, perceivers should be motivated to accurately track their partner’s boredom so that they can respond accordingly (e.g., by taking steps to maintain the relationship, or withdrawing from the unfulfilling relationship).

Perceptions of relational boredom may also be biased in one direction. That is, although Derek may accurately track changes in how Meredith feels about their relationship, he may also have a general tendency to over- or underestimate. When it comes to *relationship-threatening* perceptions, such as whether one’s partner finds the relationship unfulfilling, accurately perceiving a partner’s mean levels of that experience has the potential to undermine relationship satisfaction and stability (e.g., Simpson et al., 1995). If Meredith believes that Derek has become bored with their relationship, these beliefs may heighten her concerns about being hurt or rejected. In cases like this, where accurate knowledge of a partner’s feelings may threaten the perceiver’s self-esteem or their esteem of the relationship, both risk regulation theory (Murray et al., 2006) and the empathic accuracy model (Ickes & Simpson, 1997) suggest that people are motivated to be *inaccurate*. Guided by this idea, *underestimating* a partner’s relational boredom may be a method of self-protection, allowing the perceiver to avoid the threat of knowing their partner is bored.

Recent research has not only examined whether partners accurately perceive one another, but has also linked the pattern of their (in)accuracy to relational outcomes, such as relationship and sexual satisfaction, closeness, love, and conflict (Dobson et al., 2018, 2022; LaBuda & Gere, 2021; LaBuda, 2022; Luo & Snider, 2009). With boredom, for example, it is possible that if Meredith underestimates Derek’s boredom as a means of self-protection, she is able to maintain her relationship satisfaction to some extent even if Derek is bored. We therefore examine the links between various levels of (in)accuracy and three of the most common benchmarks of relationship quality in the existing literature (e.g., Joel et al., 2020; Le et al., 2010): relationship satisfaction, commitment, and trust.

### The Present Research

No research has examined whether accurate and biased perceptions of boredom play a role in relationship quality. We address this gap in three dyadic studies. In Studies 1–2, we tested partners’ cross-sectional experiences of tracking accuracy, directional bias, and assumed similarity (RQ1), and the associations of accuracy and bias with indicators of relationship quality (RQ2).

In Study 3, we investigated these effects in partners’ day-to-day lives. Study 3 also explored the possible motivational function of accurate and biased perceptions of relational boredom, testing whether accurate knowledge of a partner’s boredom predicted subsequent behaviours aimed at coping with that boredom (e.g., endeavouring to introduce more novelty into the relationship). Lastly, in Study 3 we explored whether partners’ boredom coping behaviours served as a cue for actual boredom, and if perceivers used that cue to inform their judgments.

## Study 1

Our first goal in Study 1 was to investigate the interplay of tracking accuracy and directional bias in partners’ perceptions of relational boredom (RQ1). We predicted that partners would demonstrate tracking accuracy in their judgments of each other’s boredom, a hypothesis consistent with prior research (Fletcher & Kerr, 2010). Guided by studies showing that people tend to project their own relationship experiences onto their perceptions of their partner (e.g., Kenny & Acitelli, 2001), we also expected that partners would assume similarity when judging each other’s boredom. Lastly, we expected that perceivers would underestimate their partner’s boredom, erring in a manner that protects the self from relationship threat (Murray et al., 2006; 2008). This prediction is also consistent with past findings demonstrating a general tendency for partners to underestimate one another’s relationship-centered behaviours, judgments, and beliefs (Fletcher & Kerr, 2010). We note that although these hypotheses were informed by risk regulation theory, we do not formally test the risk regulation model in the current research.

Our second goal was to examine how accurate and biased perceptions of relational boredom would predict perceivers’ and partners’ satisfaction, commitment, and trust (RQ2). In line with the concept of motivated inaccuracy (Ickes & Simpson, 1997), we expected that *underestimation* of boredom, as well as accuracy, would be associated with better relationship outcomes for *perceivers*. We explored the possibility that the link between directional bias and relationship outcomes would differ for perceivers’ partners, because underestimation may mean that perceivers have “missed the mark” with how their partner truly feels about the relationship. In contrast, *overestimation* of boredom may indicate to perceivers that they should enact relationship maintenance behaviours, which could promote better relationship outcomes for *partners*. However, given that it is currently unclear in the literature how one form of directional bias or the other should be linked to relationship quality for perceivers and partners, we made no firm a priori predictions and kept these analyses exploratory in Study 1.

## Method

### Transparency and Openness

Our a priori hypotheses, materials, analytic plan, data, output, and syntax appear at https://osf.io/vj87q/. Although we posted our hypotheses, materials, and analytic plan on this OSF page prior to analysis, they were not formally preregistered.

### Participants

The sample for Study 1 comprised 80 heterosexual romantic couples (all men-women couples, including cis-and transgender individuals) recruited from the London, Ontario community.^1^ Participants were 18–68 years of age (*M*_years_ = 23.64, *SD*_years_ = 8.21, Median_years_ = 20.00) and were in relationships lasting 1 month to 38 years (*M*_years_ = 2.83, *SD*_years_ = 5.33, Median_years_ = 1.25). Approximately 83% of participants were casually or exclusively dating their current partner, and 17% were common-law, engaged, or married. A minority of couples (36%) were cohabiting.

### Measures and Procedure

Data were taken from a larger cross-sectional study examining psychological experiences in romantic couples (see https://osf.io/85vmu/).^2^ Couples arrived at the lab together, where they separately and privately completed a battery of questionnaires that included the variables of interest to the present research. The study lasted approximately 90 minutes in full and was completed in a single session. After completing the study questionnaires, partners were debriefed and received CAD-$15.00 each as compensation for their participation.

#### Relational Boredom

Participants completed two versions of the Relational Boredom Scale (RBS; Harasymchuk & Fehr, 2012), a 15-item measure rated on a 7-point scale (1 = *not at all true*, 7 = *completely true*) wherein they indicated how well a series of brief descriptors characterized their relationship (e.g., “dull”; “full of surprises,” reverse-scored). In one version, participants reported their *own* levels of relational boredom (*M* = 2.22, *SD* = .88, α = .89), and in the second version they reported their perceptions of their *partner’s* levels of relational boredom (*M* = 2.28, *SD* = .88, α = .89). Each partner thus created a relational boredom profile for themselves and their beliefs about their partner.

#### Relationship Quality

Participants reported their satisfaction using the Relationship Assessment Scale (RAS; Hendrick, 1988), a 7-item measure rated on a 5-point scale (1 = *not at all/extremely poor*, 5 = *a great deal/extremely good*) that assesses how happy individuals are in their current relationship (e.g., “How good is your relationship compared to most?”). Participants reported their commitment using the commitment subscale of the Investment Model Scale (IMS; Rusbult et al., 1998), a 7-item measure rated on a 9-point scale (0 = *do not agree at all*, 8 = *agree completely*) that assesses how dedicated individuals are to their current relationship (e.g., “I want our relationship to last for a very long time”). Participants reported their trust using Rempel et al.’s (1985) 17-item measure rated on a 7-point scale (1 = *strongly disagree*, 7 = *strongly agree*) that assesses individuals’ beliefs about their partner’s dependability and honesty (e.g., “My partner has proven to be trustworthy and I am willing to let him/her engage in activities which other partners find too threatening”). Scores were calculated by averaging responses across items, with higher scores indicating greater satisfaction (α = .86), commitment (α = .89), and trust (α = .85), respectively.

## Results

We used two distinct, but complementary, statistical approaches to guide our hypothesis-testing, described in detail below.

RQ1: Patterns of Tracking Accuracy and Directional Bias in Relational Boredom Perceptions

The analyses testing our first research question were guided by West and Kenny’s (2011) Truth and Bias (T&B) Model of Judgment. Our data have a nested structure, with partners (Level 1) nested within dyad (Level 2) crossed with perceivers’ and partners’ multiple ratings of relational boredom across the 15 RBS items (because both couple members completed the same set of items). In all models couple members were treated as indistinguishable, as at the time of preregistration we did not have a theoretical rationale or hypotheses regarding what factor might meaningfully distinguish partners. In accordance with the T&B Model, perceivers’ judgments of their partner’s boredom (judgment), partner’s actual boredom (truth), and perceiver’s boredom experiences (bias) were centered on the grand mean of all partners’ boredom ratings. Using this method of centering, the intercept represents the difference between the mean of partners’ actual boredom ratings and the mean of perceivers’ judgments of those ratings, demonstrating whether their judgments differed from the partners’ actual ratings across the boredom items, and in what direction (i.e., directional bias). A *positive* average intercept indicates that perceivers generally *overestimate* partners’ boredom, whereas a *negative* average intercept indicates that perceivers generally *underestimate* partners’ boredom. The effect (slope) of the partner’s actual boredom ratings on the perceiver’s judgments of those ratings reflects tracking accuracy, and the effect (slope) of the perceiver’s own boredom ratings on their judgments of their partner’s boredom reflects assumed similarity (i.e., projection). A positive slope indicates higher tracking accuracy or assumed similarity, respectively.

As seen in the Study 1 column of Table 1, counter to expectations, perceivers marginally *overestimated* the degree to which their partner was bored with their relationship. In line with predictions, however, perceivers also demonstrated tracking accuracy and assumed similarity when making judgments of their partner’s boredom. Although not part of our original analysis plan, out of interest we ran exploratory models testing whether tracking accuracy and directional bias in perceptions of boredom varied as a function of gender, age, and relationship length. The results of these auxiliary analyses appear in our online supplemental materials.

**Table 1.** Directional Bias, Tracking Accuracy, and Assumed Similarity in Perceptions of the Partner’s Boredom.

	Study 1			Study 2			Study 3		
Perception parameter	*b* (*SE*)	CI_95%_	*R* ^2^	*b* (*SE*)	CI_95%_	*R* ^2^	*b* (*SE*)	CI_95%_	*R* ^2^
Directional bias	.07 (.04)†	[-.01, .14]	.05	.18 (.05)***	[.08, .28]	.11	.11 (.03)***	[.05, .18]	.12
Tracking accuracy	.11 (.02)***	[.07, .15]	.35	.32 (.02)***	[.28, .37]	.65	.14 (.03)***	[.08, .21]	.15
Assumed similarity	.63 (.03)***	[.58, .69]	.86	.46 (.03)***	[.41, .52]	.72	.75 (.04)***	[.67, .83]	.79

*Note*. *N*_Study 1_ = 80 heterosexual couples; *N*_Study 2_ = 136 heterosexual couples; *N*_Study 3_ = 115 heterosexual couples. We report unstandardized coefficients, with standard errors in parentheses. Approximate effect sizes were computed using the formula $R^{2}=\frac{\left(\frac{Numerator\;df}{Denominator\;df}\right)\times F}{1+\left(\left(\frac{Numerator\;df}{Denominator\;df}\right)\times F\right)}$ (see Edwards et al., 2008; Page-Gould, 2016). Degrees of freedom ranged from 62.81–80.28 (Study 1), 106.49–120.13 (Study 2), and 89.74–102.02 (Study 3).

†*p* < .10, ***p ≤ .001.

RQ2: Testing the Consequences of Accuracy and Bias in Boredom Perceptions

To test our second research question, we used multilevel polynomial regression with response surface analysis (RSA; e.g., Edwards, 2002; Nestler et al., 2019; Schönbrodt et al., 2018) following guidelines from previous research regarding the application of RSA to social psychological research (Barranti et al., 2017; Shanock et al., 2010). At the most basic level, polynomial regression with RSA examines the association of two predictors with an outcome and plots the predicted values of the outcome at all combinations of the predictors in 3D space. In some prior work, researchers have used either a categorical or interaction approach to examine the association of accuracy and bias with relationship outcomes (e.g., LaBuda & Gere, 2021). These approaches group together all levels of each type of bias (over- and underestimation) or examine effects of accuracy and bias at high (+1*SD*) and low (-1*SD*) levels only, respectively. In contrast, RSA allows us to examine predicted outcome values at all levels of accuracy and bias captured by the data. That is, these analyses allowed us to test how the degree of agreement between partners (accuracy) and how the direction of disagreement between partners (bias) were associated with relationship satisfaction, commitment, and trust.^3^ Notably, previous accuracy and bias research has also used a combination of T&B and RSA to examine the levels and impact of (in)accuracy in various domains (e.g., Dobson et al., 2018; Muise et al., 2016). Based on prior reviewer feedback and given the strong effect found for assumed similarity, we controlled for perceiver boredom (grand-mean centered) in all RSA models. We ran separate models for each relationship quality outcome.^4^ Further details regarding how to conduct and interpret RSA models can be found in our online supplemental materials.

RSA generates four surface values, designated as a_1_-a_4_. **a**_**1**_ reveals whether accuracy at high values of the predictors yield different outcomes than accuracy at low values. For Meredith’s perceptions of Derek’s boredom, a negative a_1_ would indicate that when Meredith’s perceptions and Derek’s reported boredom experiences match and are high (vs. low), levels of relationship quality are lower. **a**_**2**_ reveals whether accuracy at extreme values of the predictors yield different outcomes than accuracy at less extreme values. A significant negative a_2_ indicates that if Meredith’s perceptions match Derek’s experiences at extreme values (very high or very low boredom compared to mid-level boredom), this predicts lower relationship quality. **a**_**3**_ reveals whether one type of bias (e.g., overestimation vs. underestimation) yields different outcomes than the other type of bias. A *positive* a_3_ indicates that when Meredith *overestimates* (vs. underestimates) Derek’s relational boredom, levels of relationship quality are higher. Finally, **a**_**4**_ reveals whether accuracy yields different outcomes than inaccuracy. A significant positive a_4_ indicates that Meredith inaccurately perceiving Derek’s boredom is associated with higher levels of relationship quality than accuracy. Thus, our primary focus at the time of initial study preregistration was to examine how accuracy (represented by a_1_ and a_4_ surface values) and bias (represented by a_3_) predict relationship satisfaction, commitment, and trust. However, recent concerns raised by statisticians emphasize the limitations of interpreting surface test values independently (e.g., Humberg et al., 2019), and thus although we report all surface test values, we interpret their overall pattern through our RSA plots.^5^

**Figure 1.**
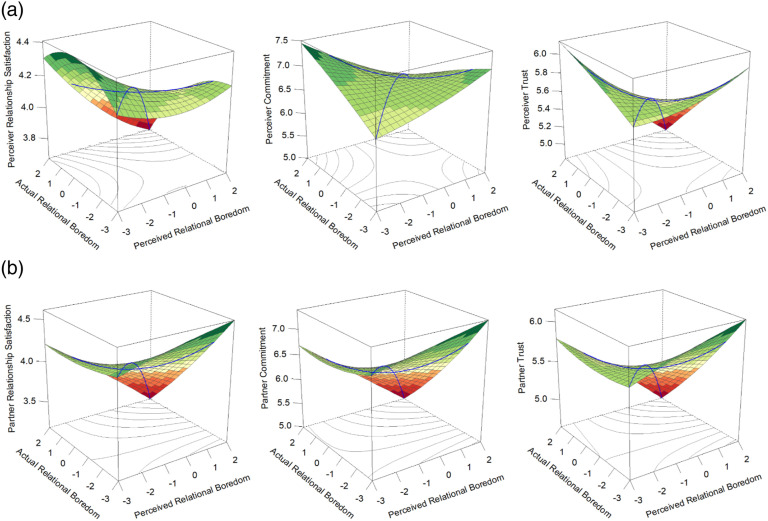
Study 1 response surface plots displaying the associations of accurate and biased perceptions of the partner’s boredom with perceiver (a) and partner (b) relationship outcomes.

The results from the RSA analyses may be viewed in Table 2 and Figures 1(a) (perceiver outcomes) and 1(b) (partner outcomes). In all studies, RSA graphs were plotted using the R package RSA (Schönbrodt & Humberg, 2021). Note that the values on the *z*-axis vary in each graph to offer the best view of the predicted 3D surface on a 2D page. However, when it did not compromise the ability to fully view the predicted surface, we endeavoured to plot the actor and partner graphs along similar *z*-axis values. Results revealed that accuracy was associated with lower relationship satisfaction, commitment, and trust than inaccuracy for both perceivers and partners (significant positive a_4_), with accuracy at high levels of boredom in particular being worse than accuracy at low or moderate levels (significant positive a_2_; significant negative a_1_, except for perceiver commitment). There were no differences in perceivers’ relationship outcomes based on whether they over- (right side of the graphs) or underestimated (left side of the graphs) their partner’s relational boredom (nonsignificant a_3,_ see Figure 1(a)). However, partners’ satisfaction, commitment, and trust were higher when the perceiver overestimated than if they underestimated (significant positive a_3,_ see Figure 1(b)). Thus, perceivers’ and partners’ relationship outcomes were generally protected if perceptions of the partner’s boredom, the partner’s actual boredom, or both, were low, and overestimation was particularly beneficial for partners’ relationship quality.

**Table 2.** Study 1: Associations of Accurate and Biased Perceptions of the Partner’s Boredom with Relationship Outcomes.

	Line of congruence		Line of incongruence	
Relationship outcome	Slope a_1_	Curvature a_2_	Slope a_3_	Curvature a_4_
Perceiver satisfaction	−.14 (.03)***	−.03 (.01)***	.02 (.02)	.02 (.01)*
Perceiver commitment	−.10 (.08)	−.07 (.03)*	−.04 (.04)	.08 (.04)*
Perceiver trust	−.22 (.04)***	−.05 (.01)***	−.01 (.02)	.07 (.02)***
Partner satisfaction	−.27 (.04)***	−.05 (.01)***	.07 (.02)**	.06 (.02)***
Partner commitment	−.45 (.09)***	−.06 (.03)*	.11 (.05)*	.11 (.05)*
Partner trust	−.27 (.04)***	−.06 (.01)***	.06 (.02)**	.07 (.02)***

*Note*. We report unstandardized coefficients, with standard errors in parentheses.

*p < .05, **p < .01, ***p ≤ .001.

## Study 2

Given that some of the results of Study 1 were inconsistent with our original theoretically-driven hypotheses, Study 2 sought to replicate Study 1 in a new sample of romantic couples. We predicted our results in Study 2 would mirror those of Study 1.^6^

## Method

### Transparency and Openness

Our a priori hypotheses, materials, analytic plan, data, output, and syntax appear at https://osf.io/krdbw/. This study’s design, hypotheses, and analytic plan were preregistered.

### Participants

Data for this study were part of a larger, two-part study conducted using Qualtrics Panel. The final sample comprised 136 heterosexual (all male-female identifying) romantic couples, who were 20–84 years of age (*M*_years_ = 48.87, *SD*_years_ = 14.58, Median_years_ = 49.00) and were in relationships lasting 7 months to 55 years (*M*_years_ = 20.66, *SD*_years_ = 14.57, Median_years_ = 1.54). Approximately 6% of participants were casually or exclusively dating their current partner, and 94% were common-law, engaged, or married. Most couples (96%) were cohabiting. Most participants identified as White (87.9%; 4.4% Black or African American; 4.0% Asian; 1.5% American Indian or Alaskan Native; 1.8% Hispanic or Latino; .4% as White and Black).

### Measures and Procedure

The data were collected as part of a larger study on romantic and sexual ideals. Couples accessed the study online and separately and privately completed a battery of questionnaires that included the variables of interest to the present research across two study sessions that were 2 weeks apart. The study lasted approximately 20 minutes per survey session. After completing the study questionnaires, partners were debriefed and received prorated compensation for their participation based on how many questionnaires they answered (up to US-$1.08 for Part 1 of the study and up to US-$1.40 for Part 2). For the present research, we use only Part 1 data.

#### Relational Boredom

Participants completed the same two versions of the RBS (Harasymchuk & Fehr, 2012) as in Study 1 (actual boredom: α = .95, *M* = 2.78, *SD* = 1.39; perceived boredom: α = .96, *M* = 2.92, *SD* = 1.44).

#### Relationship Outcomes

Participants reported their satisfaction and commitment using a subset of items from the corresponding subscales of the IMS (Rusbult et al., 1998). Satisfaction was measured with 3 items (e.g., “I feel satisfied with our relationship”) and commitment was measured with four items (e.g., “I am committed to maintaining my relationship with my partner”). All items were rated on a 9-point scale (1 = *do not agree at all*, 9 = *agree completely*). Scores were calculated by averaging responses across items, with higher scores indicating greater satisfaction (α = .94), and commitment (α = .96), respectively.

## Results

The data analytic strategy in Study 2 was identical to Study 1.

### RQ1: Patterns of Tracking Accuracy and Directional Bias in Relational Boredom Perceptions

As seen in the Study 2 column of Table 1, consistent with Study 1, perceivers significantly *overestimated* the degree to which their partner was bored with their relationship, demonstrated tracking accuracy, and assumed similarity between their own and their partner’s boredom.

### RQ2: Testing the Consequences of Accuracy and Bias in Boredom Perceptions

**Figure 2.**
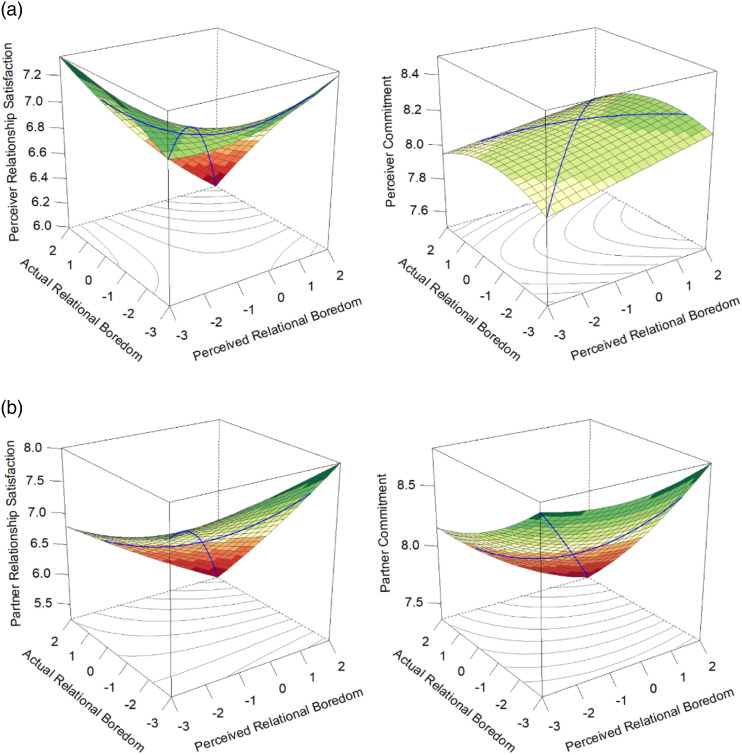
Study 2 response surface plots displaying the associations of accurate and biased perceptions of the partner’s boredom with perceiver (a) and partner (b) relationship outcomes.

The results from the RSA analyses are in Table 3 and Figures 2(a) (perceiver outcomes) and 2B (partner outcomes). Inconsistent with Study 1, there were no significant differences in perceiver commitment based on accuracy or bias. However, consistent with Study 1, accuracy was associated with lower partner commitment than inaccuracy (significant positive a_4_). Additionally, accuracy at high levels of boredom was worse than accuracy at moderate (significant negative a_2_; perceiver and partner satisfaction) or low levels (significant negative a_1_; perceiver satisfaction, partner satisfaction, and partner commitment). There were no differences in perceivers’ relationship outcomes based on whether they over- (right side of the graphs) or underestimated (left side of the graphs) their partner’s relational boredom (nonsignificant a_3_; see Figure 2(a)). However, partners’ satisfaction and commitment were higher when the perceiver overestimated than if they underestimated (significant positive a_3_). Thus, perceivers’ relationship satisfaction was protected if perceptions of the partner’s boredom, the partner’s actual boredom, or both, were low. Overestimation was particularly beneficial for partners’ relationship quality, though their relationship satisfaction and commitment were also preserved if the perceiver was accurate at low levels of boredom. However, examining Figure 2(b) it appears that, inconsistent with Study 1, partners’ relationship outcomes were not preserved to the same extent if the perceiver underestimated. In sum, Study 2 replicated all three T&B Model accuracy effects and 11 of 16 individual RSA values, including partially replicating the overall patterns of associations between accuracy and bias and relationship quality.

**Table 3.** Study 2: Associations of Accurate and Biased Perceptions of the Partner’s Boredom with Relationship Outcomes.

	Line of Congruence		Line of Incongruence	
Relationship outcome	Slope a_1_	Curvature a_2_	Slope a_3_	Curvature a_4_
Perceiver satisfaction	−.26 (.07)***	−.06 (.02)*	.02 (.06)	.07 (.05)
Perceiver commitment	.001 (.04)	−.02 (.02)	.06 (.04)	−.01 (.04)
Partner satisfaction	−.52 (.06)***	−.06 (.02)*	.24 (.06)***	.10 (.06)†
Partner commitment	−.28 (.04)***	−.003 (.01)	.13 (.06)*	.07 (.02)***

*Note*. We report unstandardized coefficients, with standard errors in parentheses.

†*p* < .10, *p < .05, ***p < .001.

## Study 3

Studies 1-2 showed that couples show tracking accuracy and directional bias in their perceptions of discrete aspects of relational boredom at a single time point. However, past researchers have theorized that relational boredom fluctuates over time (Aron & Aron, 1986; Harasymchuk & Fehr, 2010), and such fluctuations may meaningfully impact partners’ ability to accurately perceive one another’s relational boredom. The purpose of Study 3 was to determine whether romantic partners demonstrate tracking accuracy and directional bias in their perceptions of each other’s boredom over time, and whether changes in relationship quality can be predicted by changes in accuracy and bias. Our hypotheses in Study 3 were identical to those in Study 2.

In Study 3, we also sought to extend our previous findings by exploring the potential signalling function of relational boredom for perceivers and their partners. Specifically, we explored whether perceivers’ biased judgments of relational boredom were associated with them engaging in active, relationship-focused coping behaviours aimed at maintenance, such as attempting to increase novelty or communication with their partner. We also explored the reverse side of this idea; that is, whether a decrease in partners’ boredom coping behaviours acts as a cue for perceivers that not all is well within the relationship. Boredom coping behaviours likely have a reciprocal relation with boredom perceptions, as failing to engage in those behaviours is likely a cue that the partner may be bored and perceiving a partner to be bored should signal a need for coping behaviours. Considering the potentially dynamic nature of boredom over time, we tested these exploratory analyses in same-day models that investigated the signalling function of boredom on a given day, while accounting for the previous day’s coping behaviours (i.e., potential cues).

## Method

### Transparency and Openness

Our a priori hypotheses, materials, analytic plan, data, output, and syntax appear at https://osf.io/3tgp5/. This study’s design, hypotheses, and analytic plan were preregistered.

### Participants

The final sample comprised 115 heterosexual (all male-female identifying) romantic couples recruited via advertisements posted on Kijiji, Facebook, and a participant email list, in addition to flyers posted around the local community. The majority of participants completed the study while residing in Canada (95.65%; 1.74% USA, .87% Netherlands, .87% India, .87% Unknown/Did not respond to location question). Participants were 19–64 years of age (*M*_years_ = 30.78, *SD*_years_ = 8.99, Median_years_ = 28.00), mostly White (73.04%; 16.52% Asian; 5.65% Hispanic or Latino; 3.91% African American or Native American), and in relationships lasting 5 months to 26 years (*M*_years_ = 6.83, *SD*_years_ = 5.87, Median_years_ = 5.16). Approximately 42% of participants were casually or exclusively dating their current partner, and 58% were common-law, engaged, or married.

### Measures and Procedure

The data for this study were collected as part of a larger study on daily relationship and sexual experiences (see https://osf.io/42npz/).^7^ Couples accessed the three-phase study online. Phase 1 was a 30-minute background survey, Phase 2 was a daily experience period wherein partners completed 10-minute surveys each day for 21 consecutive days, and Phase 3 was a 30-minute follow-up survey that occurred 2 days following the end of Phase 2. During Phase 2, general relationship questions (including the relational boredom measures) were included on odd numbered days, and sexuality-based measures were included on even numbered days. Partners were asked to complete questionnaires separately and privately. We used shortened versions of the focal study measures in Phase 2 to reduce fatigue, increase efficiency, and minimize participant attrition (Bolger et al., 2003). After completing all three phases of the study, partners were debriefed and received prorated compensation for their participation based on how much of the study they completed (up to CAD-$35.00 per person). For the present research, we use measures and data only from Phase 2.^8^

#### Daily Relational Boredom

On odd-numbered days during Phase 2, participants completed the same two versions of the RBS (Harasymchuk & Fehr, 2012) as in Studies 1–2 (actual boredom: *Rc =* .84, *M* = 2.38, *SD* = 1.09; perceived boredom: *Rc =* .85, *M* = 2.49, *SD* = 1.18).

#### Daily Relationship Outcomes

Participants reported their daily satisfaction using four items from the RAS (Hendrick, 1988; e.g., “How satisfied are you with your relationship?”) rated on a 5-point scale (1 = *not at all/extremely poor*, 5 = *a great deal/extremely good*). Participants reported their daily commitment using 3 items from the corresponding subscale of the IMS (Rusbult et al., 1998; e.g., “I feel very attached to our relationship”) rated on a 9-point scale (0 = *do not agree at all*, 8 = *agree completely*). Participants reported their daily trust using 3 items (e.g., “My partner is dependable”) rated on a 7-point scale (1 = *strongly disagree*, 7 = *strongly agree*). Scores were calculated by averaging responses across items, with higher scores indicating greater satisfaction (*Rc* = .80), commitment (*Rc* = .90), and trust (*Rc* = .86), respectively.

#### Daily Boredom Coping

Participants completed a checklist wherein they indicated whether they had engaged in any of seven common boredom coping behaviours with their partner each day (e.g., “Tried new things with your partner”; “Watched a movie with your partner”) adapted from Harasymchuk and Fehr (2010). Scores were the summed total of coping behaviours they had engaged in that day (0–7).

## Results

The data analytic strategy in Study 3 was comparable to Studies 1–2. In Study 3, perceivers’ and partners’ ratings of their own boredom and their perceptions of each other’s boredom across the 21 diary days (mean aggregate per day; Level 1) were nested within dyad (Level 2) crossed with time to account for the fact that couple members completed surveys on the same days.

### RQ1: Patterns of Tracking Accuracy and Directional Bias in Relational Boredom Perceptions

As seen in the Study 3 column of Table 1, consistent with Studies 1–2, perceivers significantly *overestimated* the degree to which their partner was bored with their relationship and assumed similarity when making judgments of their partner’s boredom. Additionally, perceivers demonstrated tracking accuracy, indicating they accurately tracked fluctuations in their partner’s boredom over the course of 21 days.

### RQ2: Testing the Consequences of Accuracy and Bias in Boredom Perceptions

**Figure 3.**
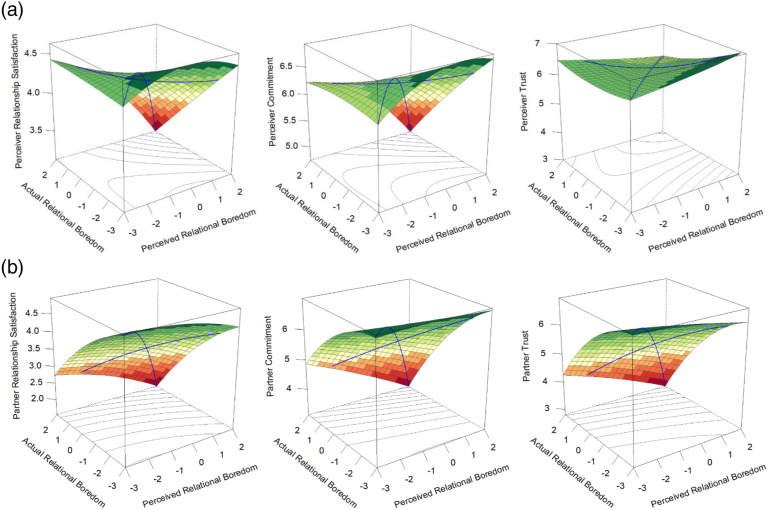
Study 3 response surface plots displaying the associations of accurate and biased perceptions of the partner’s boredom with perceiver (a) and partner (b) relationship outcomes.

The results from the RSA analyses may be viewed in Table 4 and Figures 3(a) (perceiver outcomes) and 3B (partner outcomes). Inconsistent with Studies 1–2, accuracy was only associated with lower outcomes than inaccuracy (i.e., significant positive a_4_) for perceiver trust. Accuracy and inaccuracy were associated with similar levels of perceiver satisfaction, perceiver commitment, and partner commitment (nonsignificant a_4_), and accuracy was associated with higher partner satisfaction and trust than inaccuracy (significant negative a_4_). However, consistent with Studies 1–2, accuracy at high levels of boredom was worse than accuracy at moderate (significant negative a_2_; all outcomes except perceiver trust) or low levels (significant negative a_1_; all outcomes). There were no differences in perceivers’ relationship outcomes based on whether they over- (right side of the graphs) or underestimated (left side of the graphs) their partner’s relational boredom (nonsignificant a_3_). However, partners’ satisfaction, commitment, and trust were higher when the perceiver overestimated than if they underestimated (significant positive a_3_). Thus, from Figure 3(a) we can see that perceivers’ relationship satisfaction and commitment were lower when they accurately perceived their partner’s boredom at high levels, but were protected if perceptions of the partner’s boredom, the partner’s actual boredom, or both, were low. Overestimation was particularly beneficial for partners’ relationship quality, though their relationship satisfaction, commitment, and trust were also preserved if the perceiver was accurate at low levels of boredom. However, examining Figure 3(b) it appears that, inconsistent with Study 1 but consistent with Study 2, partners’ relationship outcomes were not preserved to the same extent if the perceiver underestimated. In sum, Study 3 replicated all three T&B Model accuracy effects from Studies 1–2, 19 of 24 individual surface values from Study 1, and 11 of 16 surface values from Study 2, including the overall patterns of effects from Study 2 in particular.

**Table 4.** Study 3: Associations of Accurate and Biased Perceptions of the Partner’s Boredom with Relationship Outcomes.

	Line of Congruence		Line of Incongruence	
Relationship outcome	Slope a_1_	Curvature a_2_	Slope a_3_	Curvature a_4_
Perceiver satisfaction	−.34 (.06)***	−.09 (.02)***	−.02 (.08)	.02 (.03)
Perceiver commitment	−.44 (.09)***	−.15 (.03)***	−.08 (.12)	.02 (.05)
Perceiver trust	−.13 (.06)*	−.02 (.02)	.10 (.08)	.08 (.04)*
Partner satisfaction	−.78 (.04)***	−.11 (.01)***	.37 (.05)***	−.05 (.02)*
Partner commitment	−.93 (.06)***	−.17 (.02)***	.48 (.08)***	−.02 (.04)
Partner trust	−.97 (.06)***	−.15 (.02)***	.52 (.08)***	−.07 (.03)*

*Note*. *N*_Study 3_ = 115 heterosexual couples. We report unstandardized coefficients, with standard errors in parentheses.

*p < .05, **p < .01, ***p < .001.

### Exploratory Analyses

#### Do Accuracy and Bias in Boredom Perceptions Motivate Boredom Coping?

In Study 3, we explored the lagged effects of accuracy and bias on perceivers’ and partners’ engagement in boredom coping behaviours. Specifically, we tested whether perceivers’ overestimation of their partner’s boredom on one day would be associated with perceivers enacting greater boredom coping behaviours the following day (i.e., if overestimating a partner’s relational boredom might motivate future relationship maintenance behaviours). To test this, we used RSA as described above, while also controlling for perceiver’s engagement in boredom coping behaviours the previous day.

**Figure 4.**
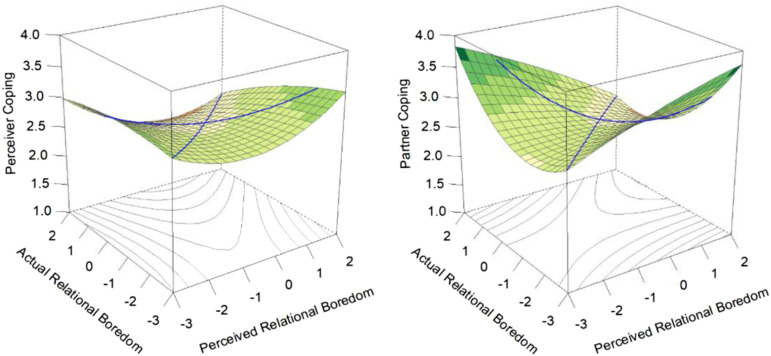
Study 3: Response surface plots displaying the associations of accurate and biased perceptions of the partner’s boredom with perceiver and partner boredom coping behaviours.

As seen in Table 5 and Figure 4, there were no effects for *perceiver* boredom coping for any surface value. For *partners*, inaccuracy (over- and under-estimation) was associated with greater boredom coping than accuracy. These findings were insufficient in explaining the consistent links found in Studies 1-3 between perceivers’ overestimation of their partner’s boredom predicting partners’ better relationship outcomes. However, given that partners’ engagement in boredom coping behaviours was associated with perceiver accuracy and bias in these analyses, we then considered the possibility that boredom coping behaviours are not a *reaction* to perceived boredom, but a *precursor* to it.

**Table 5.** *S*tudy 3: Associations of Accurate and Biased Perceptions of the Partner’s Boredom with Boredom Coping Behaviours.

	Line of Congruence		Line of Incongruence	
Relationship outcome	Slope a_1_	Curvature a_2_	Slope a_3_	Curvature a_4_
Perceiver boredom coping	−.10 (.08)	.02 (.03)	.17 (.11)	.09 (.08)
Partner boredom coping	−.10 (.08)	−.004 (.03)	−.12 (.10)	.18 (.08)*

*Note*. *N*_Study 3_ = 115 heterosexual couples. We report unstandardized coefficients, with standard errors in parentheses.

*p < .05.

#### Are Boredom Coping Behaviours a Cue Guiding Boredom Perceptions?

**Figure 5.**
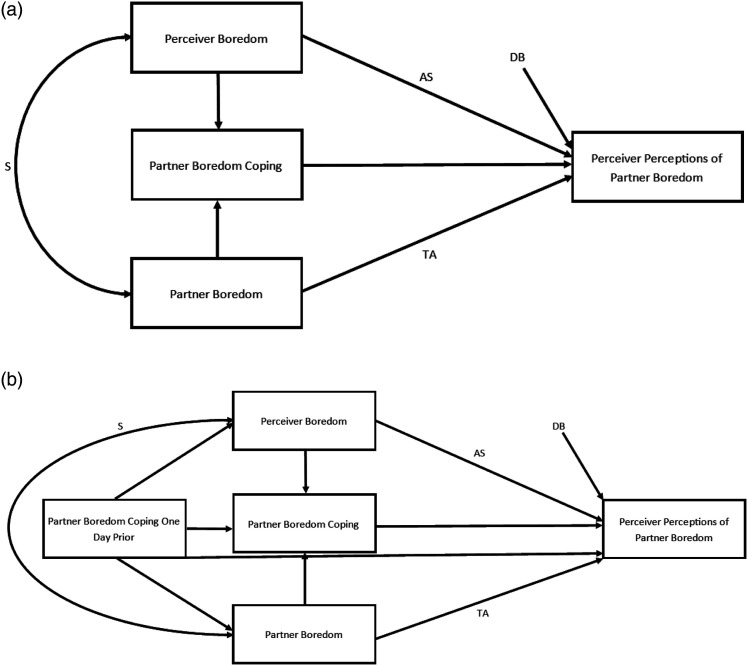
Study 3 conceptual exploratory models showing Truth and Bias estimates with partners’ boredom coping behaviours on a given day as a cue the same day (a) and behaviours on a previous day as a cue the following day (b). *Note*. S = similarity; AS = assumed similarity; TA = tracking accuracy; DB = directional bias. (B)

In our second series of exploratory analyses, we tested whether partners’ boredom coping behaviours may serve as a cue that informs perceivers’ judgments of their partner’s boredom. To explore this idea, we conducted two multilevel path models in MPlus consistent with previous accuracy and bias research (e.g., LaBuda et al., 2019). The first model tested the same-day effects of boredom coping behaviours on accuracy and bias (see Figure 5(a)), and the second model included lagged variables that tested whether boredom coping behaviours on a previous day predicted tracking accuracy and directional bias the following day (see Figure 5(b)). These models were conducted at Level 1 (day), controlling for Levels 2 (person) and 3 (couple). In these models, perceivers’ judgments of their partner’s boredom were the outcome variable, predicted by the perceivers’ own boredom and the partner’s actual reported boredom. These variables were centered around the grand mean of all partners’ boredom reports, consistent with the T&B Model (West & Kenny, 2011), to allow simultaneous testing of tracking accuracy, directional bias, and assumed similarity. We also included partners’ boredom coping behaviours to determine whether perceivers rely on their partner’s behaviours when making judgments of how bored they are with the relationship, and whether doing so improves their accuracy. If Meredith’s (perceivers’) and Derek’s (partners’) relational boredom are associated with Derek’s boredom coping behaviours, this indicates that Derek’s boredom coping behaviours are a relevant cue for how bored he is. If, in turn, Derek’s boredom coping behaviours predict Meredith’s judgments of Derek’s relational boredom, then it indicates that Meredith is indeed using these behaviours as a cue.

The results from our two models are displayed in Table 6. Findings from the first model revealed that greater partner boredom on a given day predicted fewer partner boredom coping behaviours the same day, meaning that partners’ boredom coping behaviours were a relevant cue regarding actual relational boredom experiences. Perceivers’ boredom was unrelated to partners’ boredom coping behaviours, and, interestingly, partners’ boredom coping behaviours were unrelated to perceivers’ judgments of boredom. This suggests that, although partners’ boredom coping behaviours are a relevant cue of how bored they are with the relationship, perceivers did not actually use these behaviours to inform their judgments.

**Table 6.** Study 3: Exploratory Model Results for Relational Boredom and Partner Boredom Coping Behaviours as Cues for Perceiver Accuracy and Bias.

Path	*b* (*SE*)
Model 1 (same-day)	
Similarity	.74 (.14)***
Assumed similarity	.83 (.06)***
Tracking accuracy	.18 (.05)***
Directional bias	.11 (.03)***
Perceiver Boredom→ partner boredom coping behaviours	−.10 (.07)
Partner Boredom→ partner boredom coping behaviours	−.37 (.07)***
Partner boredom coping Behaviours→ perceiver judgments of boredom	.01 (.01)
Model 2 (lagged)	
Similarity	.68 (.13)***
Assumed similarity	.84 (.06)***
Tracking accuracy	.16 (.05)***
Directional bias	.09 (.03)***
Perceiver Boredom→ partner boredom coping behaviours	−.03 (.04)
Partner Boredom→ partner boredom coping behaviours	−.22 (.05)***
Partner boredom coping Behaviours→ perceiver judgments of boredom	.01 (.01)
Partner boredom coping 1 Day Prior→ perceiver boredom next day	−.14 (.04)***
Partner boredom coping 1 Day Prior→ partner boredom next day	−.18 (.04)***
Partner boredom coping 1 Day Prior→ partner boredom coping next day	.48 (.03)***
Partner boredom coping 1 Day Prior→ perceiver judgments of boredom next day	.01 (.01)

*Note*. *N*_Study 3_ = 115 heterosexual couples. We report unstandardized coefficients, with standard errors in parentheses.

***p < .001.

Findings from the second model revealed that greater partner boredom coping behaviours the previous day predicted greater partner boredom coping and lower perceiver and partner boredom the next day. This demonstrates the enduring effects of partners’ boredom coping behaviours, and also indicates that boredom coping behaviours on a prior day were a relevant cue for boredom experiences the next day. However, as in Model 1, prior-day partner boredom coping behaviours were unrelated to perceivers’ judgments of boredom the next day, suggesting once again that perceivers did not respond to a relevant cue to their partner’s boredom.

## General Discussion

Across three studies, we examined whether romantic partners display tracking accuracy and directional bias in their perceptions of each other’s relational boredom, and whether their biased and (in)accurate perceptions were associated with relationship outcomes. Results revealed that romantic partners consistently overestimated each other’s relational boredom, displayed significant tracking accuracy both across the features that comprise relational boredom (Studies 1–2) and across time (Study 3), and assumed similarity. Additionally, accuracy and bias predicted relationship satisfaction, commitment, and trust, such that accurately perceiving high levels of boredom was associated with worse relationship quality for both perceivers and partners. However, *perceivers’* relationship quality consistently remained high if perceptions of the partner’s boredom, the partner’s actual reported boredom, or both were low. In other words, perceptions of and partner’s actual relational boredom were negatively linked to perceiver’s relationship satisfaction, commitment, and trust only when the partner’s boredom was high and the perceiver accurately recognized it as high. For perceivers, bias was consistently unrelated to relationship outcomes. The protective effects of inaccuracy for *partners’* outcomes were less consistent. For partners, accurate perceptions of high relational boredom were consistently associated with worse relationship outcomes, and overestimation and accuracy at low levels of relational boredom were consistently associated with better relationship outcomes. Underestimation, however, was associated with high levels of all partner outcomes in Study 1, only commitment in Study 2, and none of the outcomes in Study 3. Overall, overestimation (vs. underestimation) of partners’ boredom by perceivers was consistently associated with higher relationship quality for partners. Finally, we found that perceived and actual relational boredom experiences were *not* associated with perceivers’ engagement in boredom coping behaviours, and that although partners’ boredom coping behaviours are a relevant cue of boredom, perceivers may not use this cue when making boredom judgments.

Although initially we expected that perceivers would generally underestimate their partner’s boredom, erring in a manner that served a self-protective function, all three studies consistently showed that perceivers, in fact, overestimate their partner’s boredom. This is consistent with prior research on partners’ perceptions of negative interaction traits (e.g., attributes reflecting low appraisal; Dobson et al., 2022). Overestimation may serve a relationship-protective function by prioritizing the partner, a notion consistent with risk regulation theory (Murray et al., 2006; 2008). Moreover, consistent with error management theory (Galperin & Haselton, 2012; Haselton & Buss, 2000), which suggests social cognitive biases have developed over time as a means of minimizing the costs of judgmental errors, the costs associated with overestimating a partner’s boredom may be less severe than the costs associated with underestimating that boredom. The self-protective security gained by underestimating is potentially a false sense of security, putting the relationship at risk by missing signals indicating that maintenance strategies are needed. Conversely, overestimation may lead the perceiver to invest more time and resources than necessary to maintain their relationship, which may make the partner happier as they enjoy the benefits of the perceiver’s efforts. Additionally, we did not find any benefits of underestimation for perceivers or partners over and above accuracy at low levels of boredom, whereas there were consistent benefits for partners when perceivers overestimated their boredom. A general tendency to overestimate, therefore, likely reflects perceivers erring on the side of what is most beneficial to their partner and the relationship overall.

In all studies, partners displayed tracking accuracy when making judgments of each other’s boredom. Tracking accuracy likely provides the perceiver with information regarding whether they are meeting their partner’s needs, while also protecting them from investing in a relationship that their partner finds unfulfilling. Thus, the motivation to accurately track a partner’s relational boredom is likely beneficial, as it aids in both knowing when to maintain closeness and intimacy through relationship maintenance, and when there may be risk associated with maintaining the relationship, thus motivating self-protection (Murray et al., 2006). Accuracy at high levels of relational boredom was consistently associated with worse relationship outcomes for both perceivers and partners, which dovetails with previous research examining the effects of empathic accuracy on romantic relationship satisfaction and stability in relationship-threatening situations (e.g., Simpson et al., 1995). Although accuracy may offer opportunities to gain information regarding the state of a relationship, such insights in the context of a threat (e.g., when ascertaining boredom) may be painful and upsetting (Ickes & Simpson, 1997). These results also extend previous research by demonstrating that accuracy in the context of a threat is also detrimental for perceivers’ *partners*. Altogether, our findings indicate that accurately recognizing a partner’s high levels of relational boredom may impede future relationship quality for both dyad members.

Perceiver relationship satisfaction, commitment, and trust were protected if perceivers perceived their partner’s boredom as low (whether accurate or not). This is consistent with our original rationale that, as high boredom represents a relationship threat, perceiving low levels of partner boredom would serve a self-protective function. This self-protection likely yields greater feelings of security in the relationship, resulting in greater quality. Interestingly, perceiver relationship quality was also consistently preserved if their beliefs that their partner’s boredom was high were unfounded (i.e., when they overestimated boredom). Thus, overestimation of relational boredom may not invoke strong, negative emotions consistent with overestimation of other types of threats (cf. Mathews & MacLeod, 1994). Alternatively, perhaps these emotions *are* experienced in response to boredom, but boredom threatening a relationship feels manageable, with partners believing there are workable solutions to the problem in comparison to other types of threats, which protects relationship quality. Future research should examine the mechanism behind this protective effect of overestimation and examine effects over time. Underestimating a partner’s boredom may protect the self in the short-term but, as this bias is unlikely to result in corrective action, it may lead to relationship problems in the long-term. Similarly, overestimation in the long-term, and thus potentially experiencing anxiety and fear that the relationship may end, may wear perceivers down over time and cause problems later in the relationship.

Only accuracy at low levels of partner boredom and perceiver overestimation were consistently associated with higher relationship quality for partners. In Study 3, we explored whether the effects for overestimation might be because perceivers who perceive their partner is bored enact more relationship maintenance behaviours; however, we found no evidence that accuracy or bias were associated with differences in perceivers’ engagement in boredom coping behaviours. This is consistent with recent findings suggesting that although people recognize that growth-enhancing behaviours (e.g. novelty) are beneficial for combating relational boredom, these beliefs are not consistently translated into behavioural intentions (Harasymchuk et al., 2017). In fact, Harasymchuk et al. (2017) found that prescriptive beliefs translated into behavioural intentions only when competing options were made salient or the task was specific. Therefore, high perceptions of the partner’s boredom, and overestimation specifically, may predict greater boredom coping behaviours only in particular circumstances (e.g., if the partner communicates interest in an exciting activity).

It is also possible that although boredom coping behaviours may be effective, these are not the behaviours partners actually engage in when they encounter boredom. In other words, perceiving one’s partner is bored may not lead perceivers to attempt to reduce this negative experience directly, but instead create other, unrelated positive experiences that might outweigh the negative, such as increasing affection, support, or sacrifice, which also benefit the relationship (e.g., Horan & Booth-Butterfield, 2010; Impett et al., 2014). Finally, the lack of significant effects in our exploratory analyses in Study 3 may be due to our measure of boredom coping behaviours. Our measure focused on ways to reduce boredom that were rooted in the existing conceptualization of relational boredom in the literature, which primarily emphasizes understimulation (i.e., boredom resulting from having more cognitive or emotional resources available than the relationship demands). However, recent developments in general boredom research (e.g., Westgate & Wilson, 2018) suggest there are multiple profiles of boredom that occur due to deficits in the (a) level of attention required for a task and (b) meaning derived from a task. In relationships, then, partners’ boredom coping behaviours may vary based on the profile of boredom they are experiencing; for instance, Meredith’s boredom coping behaviours would likely differ for boredom resulting from overstimulation (e.g., frequent volatile arguments with Derek) than understimulation (e.g., lack of excitement). Future research should consider extending the conceptualization of relational boredom beyond understimulation and generate more nuanced measures of relational boredom and boredom coping that encapsulate various experiences.

Study 3 revealed that engagement in boredom coping behaviours is a relevant cue of partner boredom, but perceivers do not appear to use this information to inform their boredom judgments. We propose two potential reasons for this: (1) Perceivers are aware these behaviours are relevant cues for their partner’s relational boredom, but they fail to recognize when these behaviours are occurring; or (2) partners accurately perceive these behaviours but fail to recognize them as a relevant cue. Past research on coping with relational boredom suggests that when asked to reflect on which behaviours may be an effective response to boredom people often believe they should engage in growth-enhancing novel behaviours consistent with those used in the current study (Harasymchuk et al., 2017). Similarly, the boredom coping behaviours used in our research were ones identified by participants as coping behaviours in previous research (Harasymchuk & Fehr, 2010). This suggests (1) may be more likely than (2), as people appear to recognize the association of these behaviours with boredom when explicitly asked to reflect on them. However, it is possible this link is apparent to people only upon prompting and given time for reflection, and is not necessarily recognized in real time. Thus, we believe both explanations are possible given the current evidence. Both possibilities also have important implications for how to aid romantic partners in perceiving and alleviating one another’s boredom experiences and are readily amenable to future research.

This research is not without limitations. We focused specifically on perceptions of boredom as they occurred in heterosexual, monogamous romantic relationships, which limits the generalizability of our results. Recruiting couples was required in this research, but the couples who chose to participate may be happier (and less bored) with their relationships than in a sample of individuals (e.g., Park et al., 2021). Additionally, we did not collect data on class information (e.g., employment or occupational status; income; socioeconomic status, educational status), student status, or disability information in our studies, which is a limitation of this work and is thus an area for future research to consider. In addition, although we were able to examine whether partners accurately perceive fluctuations in each other’s boredom across time, the 21-day time frame for Study 3 was relatively short. It is possible that boredom fluctuates more over extended periods, with relatively small changes happening on a day-to-day basis. Future research should examine whether partners can track one another’s boredom across lengthier time periods, and across major life transitions (e.g., transition to parenthood). Finally, our interpretation of the associations of accuracy and bias with relationship outcomes assumes that accuracy and bias are, in fact, driving the effects. However, it is possible that additional factors influence the likelihood of over-versus underestimating boredom and are themselves associated with relationship quality (e.g., narcissism). Future research could consider individual or relationship characteristics that may be driving the current effects.

## Conclusion

We present three studies addressing an important gap in the relational boredom literature by examining whether romantic partners’ perceptions of each other’s relational boredom are accurate and biased, and how this (in)accuracy is associated with their own and their partner’s relationship quality. Our findings suggest that romantic partners are fairly accurate in their perceptions of each other’s relational boredom, and that accuracy and bias are associated with differences in relationship quality, but the effects of bias differ for perceivers and their partners. Understanding the reasoning behind these effects may be the next step towards helping romantic couples understand how to maximize the long-term benefits and avoid the costs of accurate and biased partner perception in relationship threatening situations.

## Supplemental Material

Supplemental Material - Are You Tired of “Us?” Accuracy and Bias in Couples’ Perceptions of Relational Boredom Click here for additional data file. Supplemental Material for Are You Tired of “Us?” Accuracy and Bias in Couples’ Perceptions of Relational Boredom by Kiersten Dobson, Sarah C. E. Stanton, Rhonda N. Balzarini, and Lorne Campbell in Journal of Social and Personal Relationships

## Notes

1 We report details on the final sample for all studies. Details on the target sample size and number of exclusions for each study are provided in our online supplemental material on the OSF.

2. Unrelated data from this study were presented in Muise et al. (2016).

3. Note that we do not refer to tracking accuracy or directional bias specifically when discussing the RSA models as there are no directly equivalent effects for these in RSA. Thus, when discussing RSA and our associated results, we use the terms accuracy (i.e., matching between perceivers’ perceptions and partners’ actual boredom) and bias (i.e., mismatching between perceivers’ perceptions and partners’ actual boredom).

4. Originally, we created and tested a composite relationship quality score by averaging the standardized scores for relationship satisfaction, commitment, and trust. However, we later had concerns about the conceptual validity of this composite score and opted instead to run separate models for each relationship quality component. One result was inconsistent across the different operationalizations of the outcome variables: for perceivers, underestimation (vs. overestimation) of the partner’s boredom was linked with higher composite relationship quality, but this effect was consistently nonsignificant when satisfaction, commitment, and trust were examined separately. Thus, in the main text of this paper we present the separate analyses for our three outcomes. We note, however, that this decision was made after hypotheses for Studies 2–3 were preregistered.

5. When Studies 1–2 were originally preregistered, some key RSA interpretation papers (e.g., Barranti et al., 2017; Humberg et al., 2019; Nestler et al., 2019) were not yet published. Thus, the interpretation of results in the main text—which reflects the more robust manner of understanding RSA when answering questions regarding accuracy and bias—may not correspond completely with the wording of hypotheses from our preregistration documents.

6. In our original preregistration, two additional hypotheses were included relating to relationship security and boredom coping behaviours. However, we opted not to test these hypotheses in Study 2 for two reasons. First, given the reinterpretation of the RSA analyses from Study 1 to consider all surface test values in tandem and the fact that the effect of underestimation on perceiver relationship outcomes was no longer significantly different from overestimation when separated into individual components (satisfaction, commitment, and trust), this meant that the additional models we planned to run were unfounded. Second, the boredom coping measure only assessed experiences in the last day, but all other measures assessed overall relationship experiences, which undermined its inclusion in analyses.

7. Unrelated data from this study were presented in Dobson et al. (2020, 2022).

8. Our original preregistration included using responses from Phase 1 and 3 questionnaires and standardizing them to be comparable to the shortened daily questionnaires. However, after further consideration and consultation with statistics experts regarding the efficacy of this choice, we altered our plan and opted to test only the Phase 2 responses in our analyses.
