# Supplementary material for: Are you tired of “us?” Accuracy and bias in couples’ perceptions of relational boredom
Source: J Soc Pers Relat. 2023 Apr 3;40(10):3091–120. doi: 10.1177/02654075231168141 (PMC10632136; doi:10.1177/02654075231168141)
Supplement: Supplemental Material - Are You Tired of “Us?” Accuracy and Bias in Couples’ Perceptions of Relational Boredom [file sj-pdf-1-spr-10.1177_02654075231168141.pdf]

Supplemental Material

Contents

Target Sample Sizes and Exclusions… **2**

Further Details on Response Surface Analysis… **3**

Differences in Accuracy and Bias Based on Gender, Relationship Length, and Age… **5**

Target Sample Sizes and Exclusions

Study 1

We originally planned to recruit approximately 100-120 romantic couples from the local university and surrounding community. Due to time constraints on the project, however, this plan was revised such that recruitment would end by a specific date, regardless of whether our target N was reached. Thus, the original sample for this study was 84 couples, but four same-gender couples were removed from analyses as there were not enough for comparison to different-gender couples, and to be consistent with the other studies reported in this paper.

Study 2

The target sample size for this study included approximately 125 couples completing both parts of the two-part study. For Part 1 of the study, 6,108 couples began the survey session. Couples were then automatically screened out and removed by Qualtrics Panel during the survey (i.e., their survey session was automatically ended and they did not see any additional questions) if one or both partners did not consent to participate ($N = 1,269$); were under 18 years of age ($N = 28$); were not fluent in English ($N = 66$); were not currently involved in a romantic relationship ($N = 1,361$); had not been together for at least four months ($N = 60$); or were not heterosexual ($N = 697$). Couples were also automatically removed by Qualtrics Panel for failing attention checks ($N = 2,099$), because they admitted to discussing their survey responses with their partner during the study ($N = 181$), or because they indicated they were unwilling to participate in Part 2 of the study ($N = 211$). Thus, in total, 5,972 couples were excluded from the study.

Study 3

The target sample size was 100 couples (200 individuals) after accounting for attrition and failure to meet inclusion criteria. This sample is consistent with previous studies using

similar methodologies (e.g., Hagemeyer et al., 2015; Meltzer et al., 2017), but included more diary days (21 days versus 14 days) and allowed for 4200 potential data points. The original sample was 130 cohabiting couples, but 15 couples were excluded from analyses because one or both partners did not consent to participate in the study (N = 5); their responses during the initial screening process did not match their survey responses for the inclusion criteria (n = 4 non-monogamous, n = 1 same-gender couple, n = 1 were not fluent in English); or because they did not complete at least three diary surveys (n = 4).

Further Details on Response Surface Analysis

In accordance with Shanock et al.'s (2010) specifications, we centered the scores for perceivers' judgments of their partner's boredom and the partner's actual reported boredom on the midpoint of the scale (i.e., 4). We then created squared versions of these variables, as well as the interaction between perceived partner boredom and actual partner boredom, and entered all five variables as predictors in our dyadic multilevel models.

The output obtained from the polynomial regression models is not interpreted directly; instead, the output is used to examine the significance of four surface test values (a_1, a_2, a_3, and a_4), which we tested by entering the five coefficients from the polynomial regression models and their standard errors into a spreadsheet provided by Shanock et al. (2010). In RSA, the *line of congruence* represents the level of relationship satisfaction, commitment, and trust when perceivers' and partners' ratings of the 15 RBS items are essentially identical. The slope of the line of congruence is captured by $\mathbf{a_1}$, which reveals whether matches at high values of the predictors yield different outcomes than matches at low values. A *positive* a_1 indicates that when perceivers' judgments of the partner's boredom and partners' actual boredom ratings are in agreement and increase, levels of the outcome are *higher,* whereas a *negative* a_1 indicates that

when perceivers' judgments of the partner's boredom and partners' actual boredom ratings are in agreement and increase, levels of the outcome are *lower*. The curvature along the line of congruence is captured by $\mathbf{a_2}$, which reveals whether matches at extreme values of the predictors yield different outcomes than matches at less extreme values. A significant positive a_2 indicates that matches at extreme values predict higher levels of the outcome compared to matches at less extreme values.

The line perpendicular to the line of congruence is the *line of incongruence*, which represents the level of relationship quality when perceivers' and partners' ratings of the 15 RBS items mismatch. The slope of the line of incongruence is captured by $\mathbf{a_3}$, which reveals whether one type of mismatch between the predictors (e.g., overestimation vs. underestimation) yields different outcomes than the other type of mismatch. A *positive* a_3 indicates that when perceivers *overestimate* (vs. underestimate) their partner's relational boredom, levels of the outcome are higher, whereas a *negative* a_3 indicates that when perceivers *underestimate* (vs. overestimate) their partner's boredom, levels of the outcome are higher. The curvature along the line of incongruence is captured by $\mathbf{a_4}$, which reveals whether matches at predictor values yield different outcomes than mismatches. A significant positive a_4 indicates that mismatches (inaccuracy) are associated with higher levels of the outcome than matches (accuracy).

The above description of surface test values indicates how each value would be interpreted if it occurred in isolation (Barranti et al., 2017), and served as the basis for our original hypotheses. However, recent concerns raised by statisticians emphasize the limitations of interpreting surface test values independently (e.g., Humberg et al., 2019). Although we recognize that the four surface test values rarely occur in isolation and must be interpreted together, to the best of our knowledge there are currently no firm guidelines on how to do this in

the context of accuracy and bias in perceivers' judgments of their partners. Thus, at present it appears to be up to the researcher to consider the size and validity of RSA effects based on theoretical consistency and prior empirical studies.

For those readers interested in a deeper understanding of dyadic RSA specifically, we recommend Schönbrodt, et al.'s (2018) paper demonstrating that RSA is a valid approach with multilevel data when the proper adjustments are made to account for the multilevel structure. The authors also provide illustrative examples and R and Mplus scripts. There are also various additional papers both within and outside relationship science that used RSA with dyadic data (e.g., Kim et al., 2021; Mund & Johnson, 2021; Weidmann, et al., 2017) and may be of interest for those looking for additional examples of its applications.

Differences in Accuracy and Bias Based on Gender, Relationship Length, and Age

Although we were skeptical regarding our power to detect such effects, out of interest and since the data was available across all studies, additional T&B models were run testing the association of gender, age, and relationship length with directional bias, tracking accuracy, and assumed similarity. These models are included in our output files for each study, and results are summarized here. In Study 1, no differences were found in directional bias, tracking accuracy, or assumed similarity based on age or relationship length. A significant effect of gender emerged for directional bias ($b(1064.64) = .07$, SE = .02, $p = .003$), and additional analyses revealed that men overestimated ($b(71.44) = .17$, SE = .06, $p = .008$) while women showed no bias ($b(78.04) =$.-.05, SE = .06, $p = .415$).

In Study 2, no differences were found in directional bias, tracking accuracy, or assumed similarity based on age or relationship length. A significant interaction of gender emerged for directional bias ($b(2035.59) = -.06$, SE = .03, $p = .048$) and assumed similarity ($b(2315.73) = -$

.08, SE = .03, p = .004), such that men overestimated (b(121.10) = .15, SE = .06, p = .008) and assumed similarity (b(3374.17) = .46, SE = .02, p < .001) to a greater extent than women, who also overestimated (b(129.70) = .13, SE = .06, p = .024) and assumed similarity (b(3161.78) = .39, SE = .02, p < .001).

In Study 3, significant interaction of relationship length emerged for tracking accuracy (b(88.82) = .01, SE = .01, p = .024), such that those who were together longer displayed significant tracking accuracy (b(92.07) = .21, SE = .04, p < .001), but those who were together for a shorter period did not (b(97.60) = .07, SE = .05, p = .139). Significant interactions of age emerged for tracking accuracy (b(151.11) = .01, SE = .003, p = .008) and assumed similarity (b(162.51) = -.01, SE = .004, p = .047), such that those who were older displayed significant tracking accuracy (b(124.69) = .22, SE = .04, p < .001) and assumed similarity (b(125.32) = .68, SE = .05, p < .001), but those who were younger did not display tracking accuracy (b(120.86) = .06, SE = .04, p = .197) and assumed similarity to a lesser extent (b(125.23) = .83, SE = .05, p < .001), although still significant. A significant interaction of gender emerged for directional bias (b(1079.72) = .10, SE = .01, p < .001) and tracking accuracy (b(1147.14) = .-.04, SE = .02, p = .036), such that men overestimated (b(112.53) = .17, SE = .05, p = .001) but women showed no bias (b(107.40) = .06, SE = .05, p = .245), and women displayed greater tracking accuracy (b(1679.38) = .16, SE = .02, p < .001) than men (b(1838.70) = .12, SE = .02, p < .001), although the effects for both were significant.
